# The British Journals: Surgery

**Published:** 1842-01

**Authors:** 


### SURGERY.

DEEP-SEATED ABSCESS OF THE BREAST. There is a disease of the breast, differing from the common milk abscess, which must, I apprehend, be of rare occurrence, having seen only four or five examples in the course of my life. All the cases which have fallen under my observation have taken place during suckling, and generally soon after delivery. It does not exactly correspond to the description of the deep-seated abscess mentioned in Mr. Hey's book, the cavities of which were filled with a soft purple fungus, although in other respects it nearly resembles it, and both are cured by the same plan of treatment.

This abscess forms in and behind the mammary gland, more slowly and with less pain than the common milk abscess; it does not come to the surface so soon, neither is the skin so much inflamed or the fluctuation so distinct, consequently it is not opened early, but burrows behind and around the gland, breaking in several places, and forming sinuses in a variety of directions, none of which show a disposition to heal. The usual treatment is unavailing in these cases; a great discharge is constantly kept up, which soon affects the general health, and reduces the patient to a state of great debility, which I believe would destroy her, unless proper means are resorted to, to arrest the disease. The first case of the kind which fell under my care was that of a lady who had been confined three months, and was much exhausted by pain and constant discharge. The breast, which was naturally full and fleshy, was not much increased in size, but had an unhealthy appearance, with several openings leading to deep sinuses, communicating with each other in various directions. At this time, I was not aware of the obstinate nature of the disease, and endeavoured to heal these by injections of sulphate of zinc, stimulating frictions, and pressure evenly applied with plaster and bandage; but all my efforts were ineffectual, and I was under the necessity of dividing all the sinuses, which ran through the whole substance of the breast, so that it was literally cut into strips. The operation was a serious one, and attended with considerable hemorrhage, but the good effects were presently apparent, for as soon as my patient had recovered from the immediate shock of the operation, the wounds assumed a healthy aspect; she rapidly got better, and recovered perfectly in six weeks. At her next confinement she came from a considerable distance to place herself under my care, and as there was no hope of her ever being able to furnish milk again from that breast, I covered it carefully with a diachylon plaster as soon as she was delivered, which completely suspended the secretion of milk in that breast, although she was able to nurse as well as usual in the other, and she passed through her confinement without the least inconvenience. Since that time she has had several other children, and uniformly adopted the same course with the like success. I have met with three other cases which were treated in the same manner, with the same result. I was consulted on another case, in which the patient refused to submit to the plan proposed, and tried various remedies under different practitioners, but was at last obliged to have the same operation performed. If I saw one of these cases in an early stage, I should not hesitate to make a free opening the instant the formation of matter could be ascertained, in the hope of preventing so painful and severe an operation.—Mr. TOOGOOD, *Provincial Medical and Surgical Journal,* No. xlvii. *Aug.* 21, 1841.

MR. LISTON'S ISINGLASS PLASTER. Mr. Liston has for many years been in the habit of using, after operations and for other surgical purposes, a plaster, consisting of oiled silk covered with a coating of isinglass. This plaster is mentioned in his well-known work on Practical Surgery, (third edition, p. 35.)

The following is the method of preparing it. Moisten an ounce of isinglass with two ounces of water, and allow it to stand for an hour or two until quite soft; then add three ounces and a half of rectified spirit, previously mixed with one ounce and a half of water. Plunge the vessel in a saucepan of boiling water, and the solution will be complete in a few minutes.

Having stretched the oil silk on a board, by nailing it round the edges, apply the solution of isinglass with a brush, taking care to move the brush evenly and in the same direction, making it smooth as you proceed; as in varnishing a picture. When quite hard and dry, apply another layer, in the same manner, but moving the brush in the opposite direction, in one case horizontally, in the other perpendicularly. In this manner apply four coats of the solution, or even a fifth, if the surface be not entirely smooth. The last layer should be reduced in strength by the addition of a little more water and spirit. An ounce of isinglass is sufficient for about a square yard of the plaster.

The following precautions should be observed: the distance between the nails should not be more than an inch and a half, otherwise the oil silk will shrink in festoons and will not remain flat. The isinglass must be well soaked in water before the spirit is added, otherwise it will not make a complete solution; and the spirit, when added, must be diluted with a portion of the water, to prevent precipitation of the isinglass The brush must be a flat "hog tool," such as is used for spirit varnish, and well made, otherwise the hairs will be found to come out, and this is an inconvenience, as the operation must be performed quickly while the solution is warm. The solution, when cold, should be of the consistence of blancmange.

Repeated experiments have shown that gelatine does not answer the purpose as a substitute for isinglass, either alone or in any proportions. A sample is on the table, in which the first three layers were composed of a mixture of the two substances, and the last is pure isinglass; in another sample only isinglass was used, and it will be seen that the latter is much more adhesive.

The oil has been, in a great measure, superseded by the use of a membrane, consisting of the peritoneal covering of the *cæcum* of the ox, rubbed down and carefully polished in the manner in which the common goldbeater's skin is prepared.. The following is the report of this plaster, furnished by Mr. Ancram, Mr. Liston's assistant at the North London Hospital.

"From the extreme thinness of the plaster, the wounds can be examined without its removal. It adheres much better than plaster made with isinglass spread on silk; and in the first instance of its application becomes firmly fixed. It is difficult to fix the isinglass plaster spread on silk, unless it is very good. From the extreme tenuity of the membrane plaster, it is equally unirritating with goldbeater's skin; and when once applied it remains so accurately adherent, that it does not require to be changed for many days. Altogether, after a good deal of experience in all the different plasters, we find it the best uniting material that has ever been produced."

In applying the isinglass to the membrane plaster, the directions already mentioned, with reference to the oil silk, may be observed, but a layer of drying oil is spread on the other side of the membrane.—Mr. Jacob Bell, *in Pharmaceutical Transactions*, No. iv, *Oct.* 1841.

Complete Dislocation of the Fifth Cervical Vertebra, without Fracture. Sir Astley Cooper, in his work on Dislocation, appears to have doubted the possibility of a complete dislocation of a vertebra without fracture: and Boyer, Delpech, and others maintained absolutely that such a coincidence could not possibly happen. But the contrary is established by cases recorded by Mr. Lawrence, Sir C. Bell, Professor Rust, and Ehrlich, as well as by the case which forms the subject of this paper. This case, however, differs from those adverted to, in having taken place in a backward and downward direction; all the other cases, on record, having taken place forwards and upwards. It is extremely remarkable and interesting that the same individual, to whom this fatal accident occurred, besides presenting a perfect anchylosis of the five superior cervical vertebræ, had had a former dislocation, by which the "atlas was pushed upwards and forwards from off the articulatory surface of the axis, so as to cause the odontoid process to present itself nearly in the centre of the circle of the atlas. A bridge of bone, exactly half an inch in length, and varying from three to four lines in breadth, passes nearly horizontally forwards, from the odontoid process to the atlas, as described above; and connects them together."—*Case by* S. S. Stanley, Esq., Assistant Surgeon of the Royal Hospital, Haslar; *in Edinburgh Medical and Surgical Journal, Oct.* 1, 1841.

